# Melanoma-specific MHC-II expression represents a tumour-autonomous phenotype and predicts response to anti-PD-1/PD-L1 therapy

**DOI:** 10.1038/ncomms10582

**Published:** 2016-01-29

**Authors:** Douglas B. Johnson, Monica V. Estrada, Roberto Salgado, Violeta Sanchez, Deon B. Doxie, Susan R. Opalenik, Anna E. Vilgelm, Emily Feld, Adam S. Johnson, Allison R. Greenplate, Melinda E. Sanders, Christine M. Lovly, Dennie T. Frederick, Mark C. Kelley, Ann Richmond, Jonathan M. Irish, Yu Shyr, Ryan J. Sullivan, Igor Puzanov, Jeffrey A. Sosman, Justin M. Balko

**Affiliations:** 1 Department of Medicine, Vanderbilt University, Nashville, 37232 Tennessee, USA; 2 Department of Pathology, Microbiology and Immunology, Vanderbilt University, Nashville, 37232 Tennessee, USA; 3 Department of Pathology, Breast Cancer Translational Research Laboratory, Institut Jules Bordet, Boulevard de Waterloo 121, Brussels 1000, Belgium; 4 Department of Cancer Biology, Vanderbilt University, Nashville, 37232 Tennessee, USA; 5 Department of Veterans Affairs, Tennessee Valley Healthcare System, Nashville, 37232 Tennessee, USA; 6 Department of Medicine, Massachusetts General Hospital, Boston, 02114 Massachusetts, USA; 7 Department of Surgical Oncology, Vanderbilt University, Nashville, 37232 Tennessee, USA; 8 Department of Biostatistics, Vanderbilt University, Nashville, 37232 Tennessee, USA

## Abstract

Anti-PD-1 therapy yields objective clinical responses in 30–40% of advanced melanoma patients. Since most patients do not respond, predictive biomarkers to guide treatment selection are needed. We hypothesize that MHC-I/II expression is required for tumour antigen presentation and may predict anti-PD-1 therapy response. In this study, across 60 melanoma cell lines, we find bimodal expression patterns of MHC-II, while MHC-I expression was ubiquitous. A unique subset of melanomas are capable of expressing MHC-II under basal or IFNγ-stimulated conditions. Using pathway analysis, we show that MHC-II(+) cell lines demonstrate signatures of ‘PD-1 signalling', ‘allograft rejection' and ‘T-cell receptor signalling', among others. In two independent cohorts of anti-PD-1-treated melanoma patients, MHC-II positivity on tumour cells is associated with therapeutic response, progression-free and overall survival, as well as CD4^+^ and CD8^+^ tumour infiltrate. MHC-II^+^ tumours can be identified by melanoma-specific immunohistochemistry using commercially available antibodies for HLA-DR to improve anti-PD-1 patient selection.

## Abstract (web summary)

Immunotherapy is used to treat melanoma, however patient responses vary widely highlighting the need for factors that can predict therapeutic success. Here, the authors show that MHC-II molecules expressed by tumour cells are positively correlated with a good response to therapy and overall patient survival.

## Graphical abstract

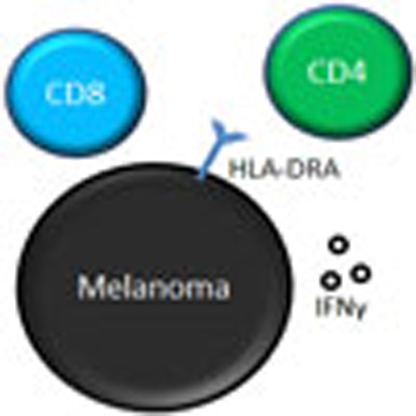

Monoclonal antibodies blocking the programmed death-1 (PD-1) receptor or its ligand (PD-L1) relieve the suppression of anti-tumour immune responses in a variety of cancers. Durable remissions occur in sizable fractions of patients with melanoma (30–40%)^1^,^2^,^3^,^4^,^5^,^6^, non-small cell lung cancer (15–20%)^1^,^3^,^7^,^8^,^9^, renal cell carcinoma (20–30%)^1^,^3^,^10^, bladder urothelial carcinoma (30%)^11^, Hodgkin's lymphoma (80–90%)^12^, and others including head and neck squamous-cell carcinoma and triple-negative breast cancer^3^,^13^,^14^,^15^. Accurate predictive markers of therapeutic efficacy are needed to optimize patient selection, improve treatment decision-making and minimize costs. To date, several candidate approaches have been identified in melanoma. These include tumour or immune cell expression of PD-L1 (refs 1, 3), identification of neoantigens through next-generation sequencing techniques^16^,^17^ and T-cell receptor clonality profiling^18^. While quite promising, these assays are technically challenging and require specialized tissue processing.

Tumours evade immune surveillance by immune checkpoint expression (PD-L1 and others), immunosuppressive cytokine profiles, tolerogenic immune cell recruitment (regulatory T-cells and others) and cancer-specific cell signalling^19^,^20^,^21^. In addition, cancer cells can lose the ability to present tumour antigens, thus avoiding recognition by cytotoxic T cells and antigen presenting cells, thus avoiding recognition by cytotoxic T cells and antigen-presenting cells (APCs)^22^. Downregulation of major histocompatibility complex class-I and -II (MHC-I and MHC-II) has been linked to immune suppression, metastatic progression and a poor prognosis in numerous malignancies^22^,^23^,^24^,^25^,^26^.

Despite the established importance of tumour-specific antigen expression, the influence of MHC-I and MHC-II expression on response to new immune therapies, particularly anti-PD-1/PD-L1, has not been explored. Specifically, HLA-DR is frequently expressed on melanoma and has unclear functional and prognostic significance^27^,^28^,^29^. We hypothesized that MHC-I and MHC-II expression, particularly HLA-DR, are required for anti-PD-1/PD-L1 activity and serve as technically and clinically feasible predictive biomarkers for therapeutic efficacy. In this study, we find that melanoma-specific expression of HLA-DR marks tumours with unique inflammatory signals that are more responsive to PD-1-targeted therapy. On the basis of this, we propose use of tumour-specific HLA-DR expression as a potential biomarker of high likelihood of response to these agents in clinical trials.

## Results

### MHC-I and MHC-II expression in melanoma cell lines

On the basis of the known biological interactions of PD-1/PD-L1-signalling, antigen presentation by tumour or professional APCs is hypothesized to be a requirement for immune recognition of the malignant cell. MHC-I presents antigen to CD8^+^ cytotoxic T lymphocytes (CTL) and is ubiquitously expressed by most cells. Loss of MHC-I is typically thought to trigger natural-killer cell checkpoints, resulting in natural-killer cell-mediated cytotoxicity. In contrast, MHC-II, which presents antigen to CD4^+^ T-helper cells, is typically restricted to professional APCs such as dendritic cells and B cells. HLA-DR, the primary antigen-presenting molecule of the MHC-II pathway is expressed in some cancers, particularly in response to CTL-secreted interferon-gamma (IFNγ). Some data suggest that non-immune cells, including cancer cells, can function as MHC-II^+^ APCs^30^,^31^,^32^. Given the heterogeneity of the tumour milieu, we asked whether MHC-I and II were expressed in *in vitro* cell line models of melanoma (rather than in resected melanoma tumours), where the contribution of stromal and infiltrating immune cells could be excluded.

**Figure 1:**
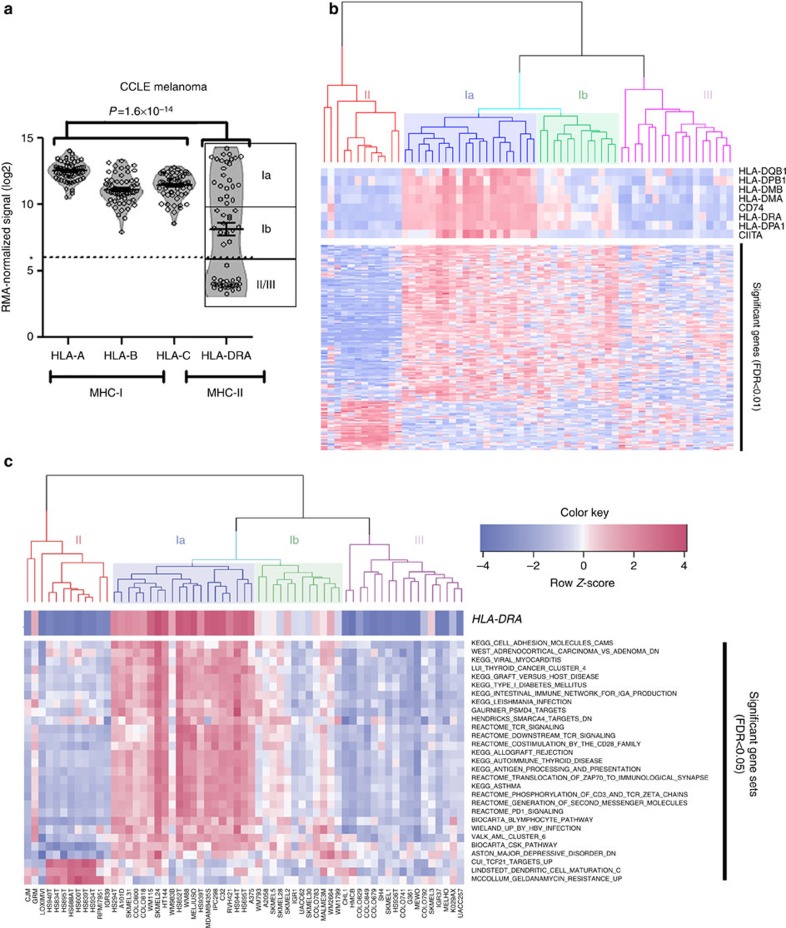
A unique subtype of melanoma expresses MHC-II. (**a**) Microarray data from 60 melanoma cell lines in the CCLE^48^ were analysed for MHC-I (*HLA-A/B/C* and MHC-II (*HLA-DRA*) expression. Bars represent the mean±s.d. *P* value is the result of the Kolmogorov–Smirnov test comparing the distribution of MHC-I (*HLA-A*, *HLA-B*, *HLA-C*) expression with MHC-II expression (*HLA-DRA*). *represents the cutoff for defining MHC-II(+). (**b**) Gene-expression data from *HLA-DRA*(+) cell lines (Clusters Ia/Ib) were compared with *HLA-DRA*(−) cell lines (Clusters II and III) by an FDR-corrected row *t*-test. Significantly altered genes are shown on the *y*-axis and also listed in Supplementary Data 1. An *ad hoc* heat map is shown at the top, highlighting classical MHC-II genes. (**c**) Normalized microarray data were analysed by GSA^47^ using the curated Molecular Signatures Database, and the resulting gene set scores are presented as a hierarchical clustered heat map.

Using the Cancer Cell Line Encyclopaedia (CCLE) melanoma panel of 60 cell lines, we determined that MHC-I mRNA expression (using *HLA-A* as the prototype) was ubiquitously high across almost all melanoma cell lines (Fig. 1a). In contrast, *HLA-DRA*, the prototype MHC-II molecule, demonstrated a strong bimodal distribution pattern, and appeared absent in ∼50% of cell lines (Fig. 1a). The remaining cell lines demonstrated intermediate-to-high mRNA levels. When cell lines were factored according to *HLA-DRA* mRNA (using an arbitrary cutoff of 6 (RMA log2 signal intensity), there was a signature of 159 genes (Supplementary Data 1) which were significantly altered (up or downregulated, false-discovery rate (FDR)<1%) in *HLA-DRA*-expressing cells compared with those cell lines lacking *HLA-DRA* mRNA (Fig. 1b). Clustering on these genes suggested four clusters of expression patterns, which we identified as clusters Ia and Ib (predominantly HLA-DR-expressing) and clusters II and III (predominantly HLA-DR-negative). Gene set analysis (GSA) of the CCLE based on MHC-II classification yielded 27 gene sets with upregulated scores and 1 with a downregulated score at an FDR≤5% in the Ia/Ib subtype. Bioinformatics analysis of the enriched gene sets suggested that *HLA-DRA*-expressing cell lines harboured expression signatures of ‘PD-1 signalling', ‘T-cell receptor signalling', ‘graft-versus-host disease' and ‘allograft rejection' (Fig. 1c). These findings suggested that there were tumour-cell autonomous signalling pathways driving MHC-II expression consistent with a pro-immune/anti-tumour response. The presence of a high mutational burden and resulting neoantigens has been shown to predict response to PD-L1 therapy in lung cancer^33^. HLA-DR-expressing melanoma lines had a higher total nonsynonymous mutational load by targeted next-generation sequencing of 1,561 genes, although this was not statistically significant (Wilcoxon rank sum test *P*=0.056, Supplementary Fig. 1).

**Figure 2:**
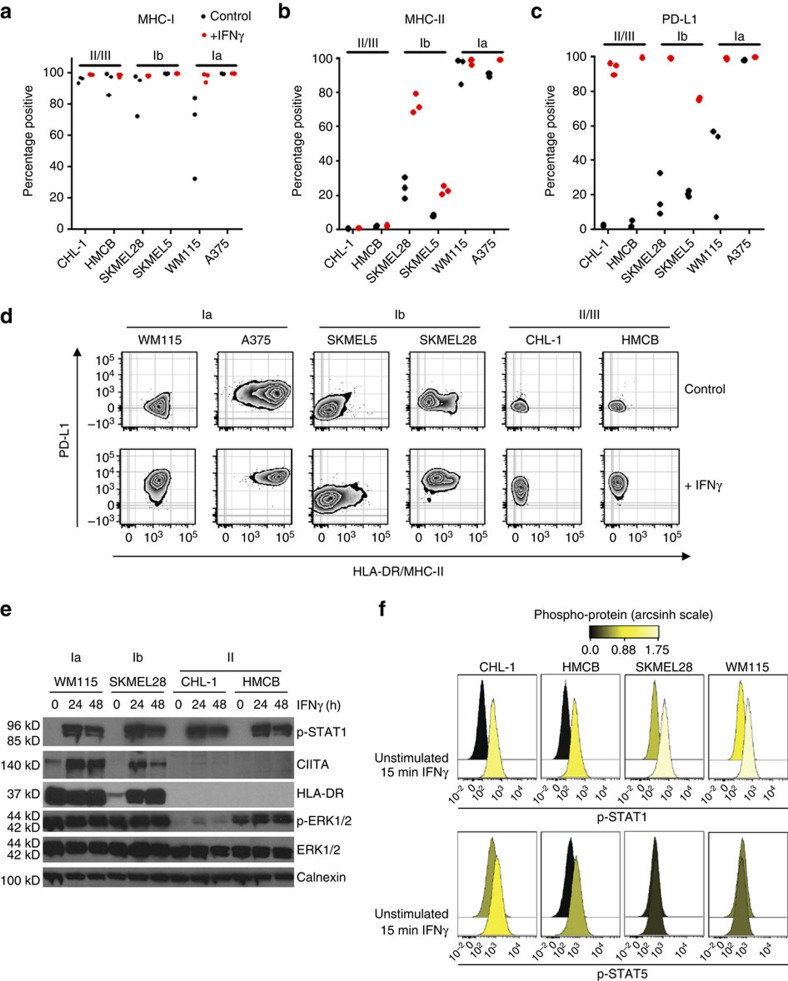
Characterization of MHC-II(+) melanoma cell lines. Melanoma cell lines were treated with IFNγ for 24 h before collection and live-cell staining and flow cytometry analysis for MHC-I/HLA-A/B/C (**a**), MHC-II/HLA-DR (**b**) and PD-L1 (**c**). Bars represent mean±s.e.m. for at least three experiments (**d**) Representative flow plots from **c**. (**e**) Western blot analysis of melanoma cell lines after 24 or 48 h of IFNγ stimulation. (**f**) Phosphorylation of STAT1 (top row) and STAT5 (bottom row) in melanoma cell lines at 15 min after IFNγ stimulation. Histograms were coloured according to the arcsinh transformed ratio or MFI medians relative to the table minimum value.

Since mRNA expression does not imply functional protein expression, and because micro-environmental IFNγ is known to influence MHC-I, MHC-II and PD-L1 expression, we characterized representative cell lines from *HLA-DRA*-expressing (cluster Ia and Ib, Fig. 1b) and *HLA-DRA*-deficient (cluster II, Fig. 1b) subgroups by flow cytometry under basal and stimulated (IFNγ) conditions. Cell-surface expression mirrored mRNA-expression patterns; MHC-I (HLA-A/B/C) expression was detected in all cell lines under both basal and stimulated conditions. However, the antibody utilized to assess MHC-I expression is reactive with all class-I alleles and haplotypes and specific class-I allele expression was not assessed in this study. In contrast, MHC-II (HLA-DR) was present only on the intermediate/Ib (SKMEL5 and SKMEL28) and high/Ia cell lines (WM115 and A375; Fig. 2a–c and Supplementary Fig. 2). No significant increase in HLA-DR expression was observed with either CHL-1 or HMCB even after 72 h of IFNγ treatment (Supplementary Fig. 2D) Notably, the intermediate/Ib cell line SKMEL28 had a unique population (25%) of cells that was constitutively HLA-DR-expressing at baseline, and was potently induced by IFNγ (Fig. 2d). The high (Ia) WM115 cell line was essentially 100% positive for HLA-DR at both basal and stimulated conditions.

Interestingly, PD-L1 expression was potently induced with stimulation in all cell lines, though the HLA-DR^+^ cell lines exhibited greater populations of cells that were PD-L1 positive in the absence of IFNγ (Fig. 2c,d). Consistent with this, STAT1 was robustly activated with IFNγ stimulation in all cell lines, whereas CIITA expression, a master regulator of MHC-II transcription, was only induced in HLA-DR^+^ Ia/Ib cells (Fig. 2e). Phospho-flow analysis demonstrated that while STAT1 was activated robustly with short-term (15 min) IFNγ stimulation, STAT5 was preferentially activated by IFNγ in MHC-II(−) cell lines (Fig. 2f), consistent with the observations of others that STAT5 can contribute to resistance to interferon signalling and phenotypes^34^. Together, these results suggest that there is a tumour-cell autonomous inflammatory signal present in a subset of melanomas that may predispose the tumour to enhanced MHC-II expression, antigen presentation (direct or cross presentation via exosomes^35^) to CD4^+^ T-helper cells and immune recognition, coinciding with higher PD-L1 expression. Furthermore, these data suggest that STAT5 activation may contribute to suppression of this inflammatory signal. Thus, we reasoned that the HLA-DR-expressing subtype of melanoma can be unmasked to the immune system by therapeutic inhibition of the PD-1/PD-L1 axis.

### HLA-DR expression by genotype

**Figure 3:**
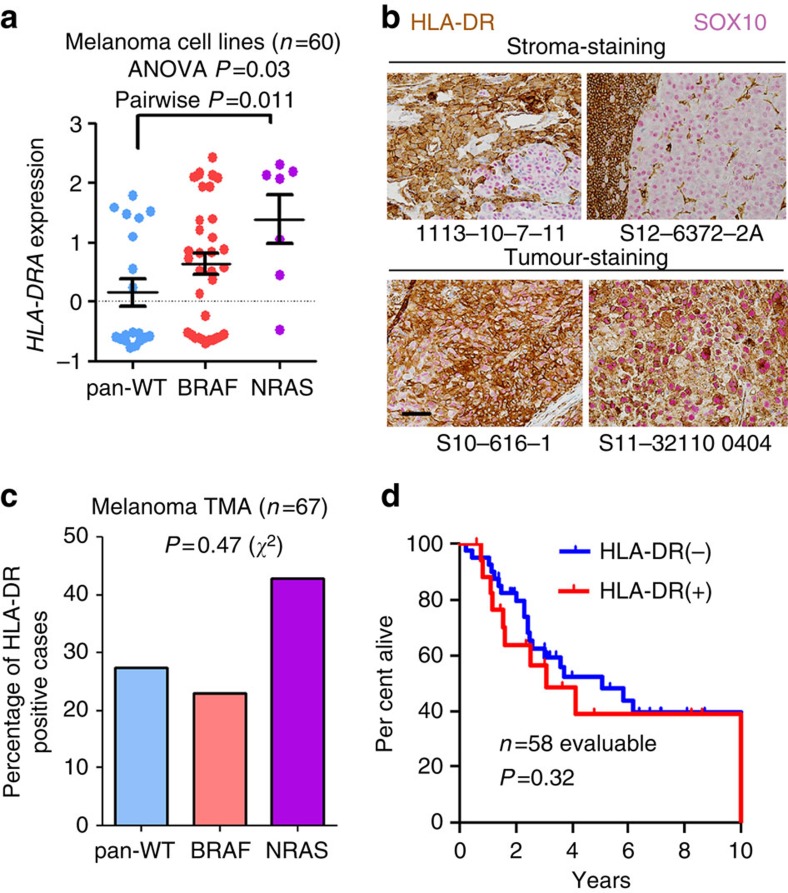
MHC-II-positive melanoma cell lines associate with NRAS mutations. (**a**) *HLA-DRA* mRNA expression in melanoma cell lines (*n*=60; one cell line lacked mRNA expression data) from the CCLE compared by genotype. *P* value (*P*<0.05) represents result of Tukey's *post hoc* analysis comparing pan-WT with NRAS-mutant cell lines, following a significant ANOVA (*P*=0.03) performed among all groups. Bars represent mean±s.e.m. (**b**) Representative IHC for HLA-DR (brown) and SOX10 (pink) in cases with isolated stromal positivity (top) and with tumour-specific staining (bottom). Both HLA-DR and SOX10 immunostaining is present in all four sections. Scale bar, 50 μm. (**c**) Analysis of HLA-DR IHC in a melanoma TMA (*n*=67 evaluable) by genotype. *P* value represents result of a *χ*^2^-test. (**d**) Overall survival of patients (*n*=58 evaluable) within the TMA by HLA-DR status (left censored at time of diagnosis). The remaining patient samples were included from outside institutions and follow-up data were not available from those institutions. *P* value is the result of the log-rank test.

*HLA-DRA* expression was specifically enriched in cell lines harbouring *NRAS* mutations (Fig. 3a). Notably, studies by our group and others have suggested that patients harbouring *NRAS* mutations experience improved response rates to PD-1 axis therapy and other immune therapies^36^,^37^. Although the biological basis of this correlation remains to be elucidated, these results were intriguing and compatible with our hypothesis. To test whether the same association could be observed in clinical samples, we investigated MHC-II/HLA-DR expression by IHC in a tissue microarray (TMA) of melanoma patient samples (*n*=67) with known *BRAF* and *NRAS* genotypes who largely had not received immune therapy (Supplementary Table 1). Dual-colour IHC was performed with HLA-DR and SOX10 to distinguish tumour versus stromal expression of HLA-DR (Fig. 3b). We observed HLA-DR (+) tumour expression in 20/67 (30%) evaluable samples. HLA-DR was expressed more frequently in the *NRAS*-mutated cohort (43%, 6 of 14) than in *BRAF*-mutated (23%, 3 of 13) and *BRAF/NRAS* wild-type populations (28%, 11 of 39; Fig. 3c), but this was not statistically significant (*χ*^2^-test *P*=0.47). Thus, *NRAS* genotype seems to trend with HLA-DR positivity, but this association does not appear to be a significant. A larger sample size would be needed to conclusively determine whether this association is apparent or absent in patients. Importantly, in this unselected population of patients, expression of HLA-DR was not associated with overall survival (log-rank *P*=0.32), suggesting that HLA-DR expression may not be generally prognostic in advanced melanoma (Fig. 3d).

### HLA-DR expression in patients receiving anti-PD-1 or PD-L1

We previously observed that in a diverse collection of melanoma cell lines, patterns of HLA-DR expression were (i) constitutively high, (ii) heterogeneous, but inducible by IFNγ, or (iii) constitutively off. Similar patterns were observed in a cohort of unselected melanoma tumours, and thus we hypothesized that these patterns may be predictive of benefit to immunotherapy.

**Figure 4:**
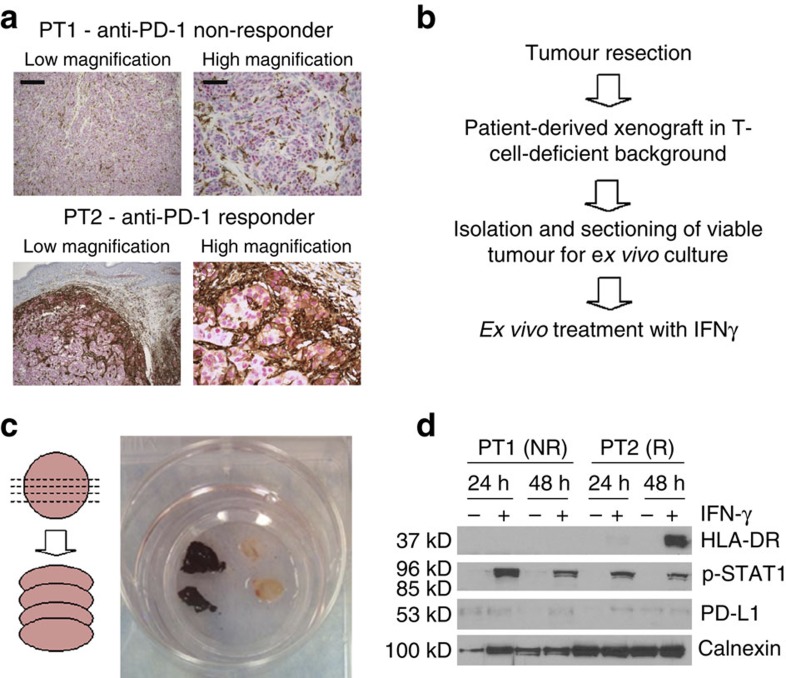
*Ex vivo* culture of tumours derived from anti-PD-1-responding and non-responding patients identifies heterogeneity in interferon response. (**a**) Patient tumour blocks stained for HLA-DR (brown) and SOX10 (pink) at low (scale bar, 500 um) and high magnification (scale bar, 200 μm); PT1: anti-PD-1 non-responder and PT2: anti-PD-1 responder. (**b**) Experimental schema. (**c**) Schema and images of PDX tissue sections (*ex vivo* organotypic culture). (**d**) Western blot analysis of tissue sections cultured in the presence or absence of IFNγ for 24–48 h.

To test this hypothesis, we used the patient-derived xenograft (PDX) models from the tumour resections of two melanoma patients who subsequently received anti-PD-1 therapy; patient 1 (PT1; non-responder, 0% HLA-DR-positive, class II/III) and patient 2 (PT2; partial responder, heterogeneous 15% HLA-DR-positive, class Ib; Fig. 4a). In PT2, the HLA-DR-staining pattern was clearly positive at the invasive interface, suggesting immune-reactivity in this particular tumour, in contrast to other tumours identified in the TMA study which were MHC-II(+) throughout the tumour. The resected tumours from PT1 and PT2 were serially transplanted to athymic nu/nu mice, which are highly deficient in functional T cells^38^, ruling out a possible source of IFNγ (Fig. 4b). Immunohistochemistry analysis of both PDX models, grown in nude mice, demonstrated no detectable HLA-DR expression. However, when PDX tumours were freshly resected, sectioned and grown *ex vivo* as cultured tissue slices, in the presence or absence of IFNγ, only the PT2 PDX model (anti-PD-1 responder) upregulated HLA-DR (Fig. 4d). Thus, HLA-DR may be a marker of IFNγ activity in the microenvironment of some (but not all) tumours. Furthermore, this experiment supports the notion that the IFNγ response varies significantly among melanomas, and demonstrates tumour-autonomous features. Furthermore, these data suggest that HLA-DR expression in melanoma cells may be a biomarker for tumours primed with activated T-cells and an appropriate IFNγ response to mediate sensitivity to PD-1/PD-L1 blockade. Importantly, however, these data do not rule out the existence of melanomas constitutively expressing HLA-DR in the absence of IFNγ stimulation, as is observed in a significant number of melanoma cell line models (Fig. 1).

In order to determine whether MHC-II expression on melanoma tumours is associated with clinical response to PD-1/PD-L1-targeted therapy, we obtained archival pre-treatment biopsy or resection specimens from 30 patients treated with anti-PD-1 (nivolumab, pembrolizumab) or anti-PD-L1 (MPDL3280A; *n*=2). The median age was 56 years, the median number of prior therapies was 1, and 14 (47%) had failed ipilimumab (Table 1). Twenty-three patients (77%) had stage IV M1c disease and 12 (40%) had elevated serum lactate dehydrogenase (LDH).

**Figure 5:**
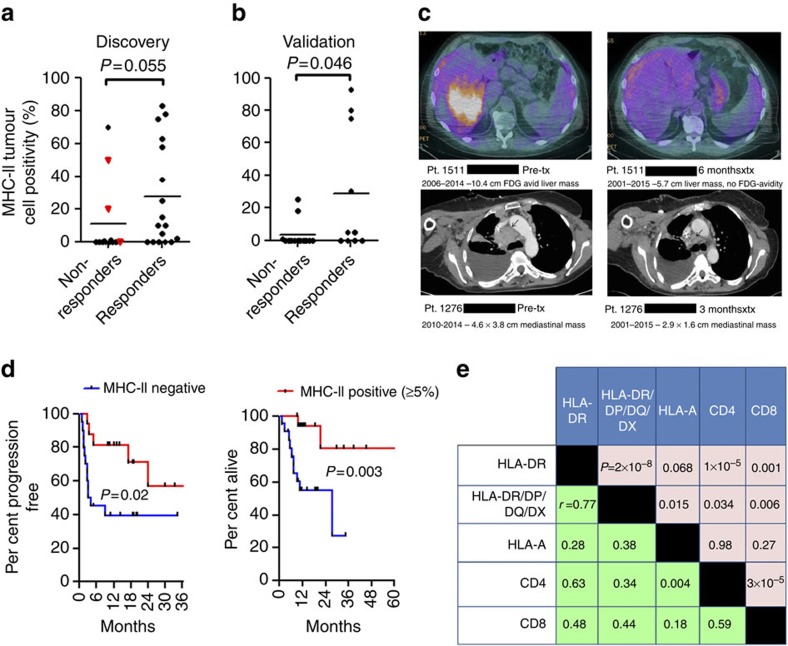
MHC-II(+) melanomas have improved response rates and clinical benefit to PD-1/PD-L1 inhibition. (**a**) HLA-DR positivity by IHC plotted versus response to PD-1/PD-L1-targeted therapy in the discovery set (*n*=30). Responders include partial and complete responders; non-responders include mixed responders and progressive disease patients. Mixed responders (*n*=3) are noted by a red triangle. *P* value is the result of the Wilcoxon's rank sum test. (**b**) HLA-DR positivity by IHC in the validation set (*n*=23) plotted versus response to PD-1/PD-L1-targeted therapy. *P* value is the result of the Wilcoxon's rank sum test (**c**) Representative images of scans from anti-PD-1 therapy-treated MHC-II(+) patients (**d**) Progression-free survival (left) and overall survival (right) in anti-PD-1/PD-L1-treated patients, stratified by HLA-DR/MHC-II positivity (5% total tumour cells staining on entire tissue section used as cutpoint). Data from both the initial and validation cohorts were included, when available. *P* value is the result of the log-rank test. (**e**) Correlation matrix of IHC markers. *P* values for the Pearson's correlation appear above the diagonal and correlation coefficients (*r*) appear below the diagonal.

We chose to differentiate MHC-II^+^ from MHC-II^−^ samples using a cutoff of >5% of tumour (SOX10^+^) membranes showing staining. Tumour HLA-DR staining strongly correlated with response to therapy. Among 14 patients with positive HLA-DR staining (>5% estimation of positive tumour membranes in the entire tissue section), 11 patients (79%) had complete (*n*=3) or partial (*n*=8) response (Fig. 5a). Clinical activity was inferior in HLA-DR non-expressing melanomas; 6 of 16 patients (38%) responded to therapy (overall response rate 79 versus 38%, Fisher's exact test *P*=0.033). Clinical benefit (including mixed responses) was similarly superior in MHC-II(+) patients (Fisher's exact test *P*=0.007). Importantly, this finding was confirmed in a second independent data set of 23 melanoma patients treated with anti-PD-1 therapy (single agent or concurrently with other immunotherapies). Of these 23 additional patients, 6/8 (75%) of HLA-DR(+) tumours responded (PR or CR), while only 4/15 (27%) HLA-DR(−) responded (Fisher's exact test *P*=0.025; Fig. 5b). Rapid objective clinical responses were observed in HLA-DR(+) tumours, even in patients with other negative prognostic features, including a patient with bulky disease, elevated LDH, impaired functional status and failure of both ipilimumab and dabrafenib/trametinib, and a patient with a >10 cm liver mass and LDH>500 U l^−1^ following failure of interleukin-2 and ipilimumab (Fig. 5c).

We also compared progression-free survival (PFS) between patient groups in both data sets, when survival data were available. The median PFS was superior in the HLA-DR (+) group (median not reached versus 3.2 months, log-rank *P*=0.02; Fig. 5d). Overall survival was also superior for the HLA-DR (+) cohort (median not reached versus 27.5 months, log-rank *P*=0.003; Fig. 5d). We excluded the three patients with mixed responses from the PFS analysis (given difficulties specifying time of clinical progression), but not the OS analysis. Importantly, statistical significance or a trend toward significance was retained at other cut-points as well, including 1, 10 and 20% (PFS log-rank *P*=0.01, *P*=0.08 and *P*=0.03, respectively, and OS log-rank *P*=0.002, *P*=0.01 and *P*=0.11, respectively; Supplementary Fig. 3). Notably, we did not observe an association with HLA-DR expression and response among 13 patients treated with ipilimumab alone, although the sample size is too small to make definitive conclusions (Supplementary Fig. 4 and Supplementary Table 2).

### MHC-II antibody specificity and concordance of assessment

To investigate the possibility of alternative MHC class II molecule expression, we performed IHC using a second monoclonal antibody targeting a common epitope to HLA-DR, -DP, -DQ and -DX (pan-MHC-II) on all samples. Results largely correlated with HLA-DR (Fig. 5e and Supplementary Fig. 5A), supporting high specificity of the HLA-DR antibody. No additional cases were identified as MHC-II(+) by use of the pan-MHC-II antibody. Pan-MHC-II positivity was also associated with objective clinical response (Mann–Whitney's *P*=0.02, Supplementary Fig. 5B) as well as PFS and OS using a 5% cut-point (log-rank *P*=0.04 and *P*=0.009, respectively; Supplementary Fig. 5C,D). Concordance in HLA-DR positivity assessment between two independent blinded pathologists was 77%. After web-mediated discussion of the discordant cases, a final consensus was reached. Concordance and consensus results of the two independent scores for HLA-DR are presented in Supplementary Tables 3 and 4, respectively.

### Other clinical correlates

To investigate the impact of MHC-I expression on response to anti-PD-1/PD-L1, we performed HLA-A IHC on the same pre-treatment samples. As observed in melanoma cell line models, HLA-A expression was nearly ubiquitous across all tumours and expression level was not statistically associated with response to therapy (Supplementary Fig. 6). However, HLA-B and HLA-C protein expression were not assessed in this study. CD4^+^ and CD8^+^ T-cell infiltration was also assessed by IHC. CD4 was not statistically associated with therapy response, while a trend towards significance was detected with CD8 (Mann–Whitney's *P*=0.077; Supplementary Fig. 7), as has been previously described^18^. The lack of statistical association in our study may be due to scoring method, as the invasive front of the tumour was not detectable in all biopsies or resection specimens. Thus, the total per cent positivity of CD8^+^ T cells invading into the tumour was calculated. Interestingly, the percentage of infiltrating CD4^+^ T cells were more strongly correlated with HLA-DR expression (Pearson's *r*=0.63; *P*=1 × 10^−5^), while CD8^+^ infiltrate was more weakly correlated (Pearson's *r*=0.48; *P*=0.001; Fig. 5e). Although HLA-DR and CD4^+^ infiltrate are biologically connected, association of HLA-DR with CD8 infiltrate may be suggestive evidence that enhanced CD4^+^ Th infiltrate could support the continued accumulation of CD8^+^ CTLs in the tumour microenvironment. In our cohort, PD-L1 immunostaining in the tumour compartment was rare, occurring in 4/24 (17%) tested patients and showed no correlation with response to PD-1/PD-L1-targeted therapy (Supplementary Fig. 8).

## Discussion

Targeting the PD-1/PD-L1-signalling axis produces durable responses in a subset of melanoma patients. Although a genetic basis for clinical response to CTLA-4 inhibition in melanoma has recently been suggested^16^, so far few studies have suggested a tumour-cell autonomous basis for response to PD-1/PD-L1 monoclonal antibodies. Herein, we have identified a unique inflammatory transcriptional signature in melanoma cell lines that can be identified by tumour cell-specific MHC-II/HLA-DR expression. Interestingly, heterogeneity in MHC-II expression among panels of melanoma lines has been previously noted^39^. We hypothesize that MHC-II expression is either (i) a functional antigen-presenting molecule that can promote CD4 T-helper cell aid to the anti-tumour milieu or (ii) a non-functional marker of the inflammatory state of the cell or tumour milieu. The presence of heterogeneity among cell lines grown *ex vivo* argues against the latter. Yet another alternative hypothesis is that MHC-II expression on melanoma cells could be instrumental in promoting Treg differentiation in a process that requires PD-1/PD-L1 interaction; thus interruption of this signalling could be beneficial in MHC-II^+^ tumours. Although we did not assess different CD4 subsets (Th1, Th2, Th17, Treg), we nonetheless observed superior clinical outcomes with anti-PD-1/PD-L1 therapy in patients harbouring melanomas with MHC-II expression.

In a bioinformatics analysis of MHC-II expression in melanoma cell lines, which rules out contaminating stromal and immune contribution, we found a number of gene-expression pathways to be upregulated in melanoma cell lines expressing MHC-II (Fig. 1c). The majority of these pathways suggested the presence of an inflammatory signature and reflected gene sets found to be upregulated in response to viral (WIELAND UP BY HBV INFECTION), parasitic infections (KEGG LEISHMANIA INFECTION) and autoimmune disease (KEGG GRAFT VERSUS HOST DISEASE, KEGG ALLOGRAFT REJECTION, KEGG ASTHMA and KEGG AUTOIMMUNE THYROID DISEASE). Biologically, these pathways reflected stimulation of T-cell receptors (REACTOME TCR SIGNALING and COSTIMULATION BY THE CD28 FAMILY) and B-cell activation (BIOCARTA BLYMPHOCYTE PATHWAY and KEGG INTESTINAL IMMUNE NETWORK FOR IGA PRODUCTION). Although several gene sets were statistically downregulated in MHC-II(+) cell lines, visual inspection of the heatmap suggested that these associations were primarily driven by high expression of target genes in a subset of MHC-II(−) cell lines, specifically Cluster II (Fig. 1c).

Although MHC-I is ubiquitously expressed in most cell types, MHC-II is typically restricted to the immune system, as the MHC-II pathway is thought to utilize extracellular antigens (released from apoptotic or necrotic cells and engulfed by professional APCs). However, tumour-specific MHC-II expression has been noted in a number of malignancies, including breast^25^, colon^23^ and melanoma^24^. Experimentally, MHC-II(+) epithelial cells can present antigen to CD4(+) T-helper cells^31^ and enforced expression of MHC-II in tumour cells can promote anti-tumour immunity and tumour rejection *in vivo*^32^. Collectively these data support a role for aberrant HLA-DR/MHC-II expressing tumours as being a uniquely immunogenic subtype (with the ability to stimulate CD4(+) T-helper cells) which may adapt by expressing PD-L1. Thus, although some MHC-II(−) tumours may express PD-L1, this alone may not permit anti-tumour immunity through PD-1/PD-L1 inhibition.

In our study, HLA-DR expression strongly correlated with response to anti-PD-1. Critically, other relevant variables also co-occurred with HLA-DR expression, demonstrated through *in silico* cell line analysis (GSA), flow cytometry of well-characterized melanoma cell lines (PD-L1 expression and CIITA expression) and pre-treatment melanoma samples (CD4 and CD8 T-cell infiltration). Together, these data strongly argue that HLA-DR plays a causal or correlative role in anti-PD-1/PD-L1 responses. Interestingly, HLA-A expression did not statistically correlate with CD8 expression in our study (Fig. 5e). This could be due to more ubiquitous expression of HLA-A among the tumours, and it could be that the spectrum of MHC-I neo-antigen may be the rate-limiting step in this association. However, MHC-II expression on the tumour did correlate with CD4 infiltrate, though the nature or composition of these CD4^+^ cells is not yet understood (Th1, Th2, Th17 or Tregs). Furthermore, in this study, only HLA-A was assessed for MHC-I. Additional contributing effects of HLA-B and HLA-C as well as non-classical MHC-I proteins were not assessed in this study due to limitations in robust antibodies and amount of tissue available for analysis.

Although our data point towards a functional role of MHC-II expression as contributing to sensitivity to PD-1/PD-L1 axis inhibition, it is important to note that some tumours responded to PD-1-targeted therapy, despite having no detectable MHC-II expression. There are several possible explanations for this observation: (i) that tumour sampling heterogeneity limited our ability to detect HLA-DR in the tumour and/or (ii) that these tumours may be similar to the Ib (interferon-inducible) group and PD-1 inhibition in these patients may increase CD8 infiltration and local IFNγ secretion, inducing HLA-DR, which could be detected by an on-treatment assessment. Of course, this is hypothetical, and also assumes that HLA-DR is a functional biomarker, rather than a surrogate, which remains to be experimentally proven. Yet a third hypothesis would be that other inflammatory/antigenic factors mediated by MHC-I (such as mutational burden and neo-antigen presence) could be sufficiently high in some cases to circumvent or abrogate an MHC-II requirement. Nonetheless, the potential role of MHC-II as a surrogate biomarker for response cannot be overlooked.

In order to demonstrate a functional role of MHC-II in promoting response to PD-1/PD-L1 therapy, we overexpressed *Ciita* in B16/F0 melanoma cells to determine whether constitutive tumour-cell MHC-II expression would enhance response to PD-L1 mAB *in vivo*. Despite previous reports^35^ of successful constitutive MHC-II (IA/IE) expression by lentivirus-mediated *Ciita* overexpression, we were unable to establish a stable population of MHC-II^+^ cells in culture, despite repeated rounds of selection and flow sorting (Supplementary Fig. 9A). Expansion of the positive population in cell numbers sufficient for the experiment routinely caused the MHC-II^+^ population to degrade to ∼1–2% after 3–5 passages. The reason for this selection is presently unclear but is a matter of current investigation. Possible explanations are silencing of the lentiviral promoter or cell-mediated internalization of MHC-II.

Nonetheless, we injected either control (*LacZ*-expressing) or *Ciita*/MHC-II^+^ B16 cells (ranging from 10 to 30% MHC-II^+^ at the time of injection) into the flank of C57/Bl6 mice and monitored tumour growth and survival with either IgG (isotype) control or anti-PD-L1 mAB, given twice weekly, beginning on day +1 following tumour-cell challenge. The subgroup of Ciita^+^ B16 melanoma cells with the highest degree of MHC-II positivity (30%) at the time of injection, treated with anti-PD-L1, had slower tumour formation and prolonged survival, although the effect was marginal (Supplementary Fig. 9B). We believe the observed effect may not have been robust due to unstable expression and rapid selection of *Ciit*a-transduced cells *in vitro* and *in vivo*. Interestingly, there appears to be an MHC-II^+^ dose–effect response to PD-L1 mAB (that is, 30% MHC-II^+^ responded better than 10 or 20%). While these results are difficult to interpret due to difficulty in establishing a pure cell line, we believe they do support a potential functional role of MHC-II expression in immunotherapy response.

Conflicting reports of stromal versus tumour PD-L1 staining, coupled with the lack of standardization, proprietary nature and the difficulties associated with PD-L1 as an IHC antigen have precluded the routine use of this marker in the clinic. In our study, a relatively low number of samples stained positively for PD-L1, despite appropriate positive controls (human placenta). The low proportion of samples with PD-L1 staining and lack of correlation of positivity with patient benefit reinforce the problems of using PD-L1 as a clinical biomarker. In contrast, HLA-DR can be robustly identified on tumour cells through use of dual-colour IHC using well-established commercially available antibodies. We propose that with additional validation, melanoma HLA-DR expression may be a rapidly translatable biomarker for patient stratification of PD-1/PD-L1 immunotherapy which can easily be performed in standard pathology laboratories at most institutions at low cost. This marker, if validated, could be envisioned to stratify patients towards anti-PD-1 monotherapy and away from the more toxic but potentially more clinically active combination of ipilimumab and nivolumab^40^,^41^,^42^. Furthermore, understanding the biological basis for differential MHC-II expression among melanomas may identify agents that induce MHC-II positivity and can be used in combination with PD-1/PD-L1-targeted therapy to enhance response rates.

## Methods

### Immunoblotting

Immunoblotting was performed as previously described^43^,^44^. Briefly, cells were washed in cold phosphate-buffered saline, collected and lysed in 1 × RIPA buffer (50 mM Tris (pH 7.4), 1% NP-40, 150 mM NaCl, 1 mM EDTA, 0.1% SDS, 0.25% sodium deoxycholate, 5 mM NaF, 5 mM Na3VO4, 10% glycerol, 1 M phenylmethyl-sulphonylfluoride and protease inhibitors) for 30 min on ice. Lysates were sonicated for 2–3 s to shear DNA and cleared by centrifugation at 13,200 r.p.m. for 15 min. Protein concentrations of the lysates were determined by BCA assay (Bio-Rad, Hercules, CA). Samples were separated by SDS-PAGE and transferred to nitrocellulose membrane. Membranes were blocked with 5% non-fat dry milk or 5% bovine serum albumin in tris-buffered saline with 0.1% Tween-20 for 1 h at room temperature and then incubated overnight at 4 °C with the appropriate antibody as indicated. Following incubation with appropriate horseradish peroxidase-conjugated secondary antibodies, proteins were visualized using an enhanced chemiluminescence detection system. This study was performed using the following antibodies: p-STAT1 (Cell Signaling Technology, #7649, 1:5,000) STAT1 (Santa Cruz Biotechnology, #SC592, 1:5,000), p-ERK1/2 (Cell Signaling Technology,#9101, 1:5,000), ERK1/2 (Cell Signaling Technology #9102, 1:5,000), CIITA (Cell Signaling Technology #3793, 1:1,000) HLA-DR (Santa Cruz Biotechnology, sc-53319, 1:5,000).

### Standard flow cytometry

Flow cytometry was performed using the following antibodies: HLA-DR/PE-Cy7 (Biolegend, clone L243, 1:20), CD274/PD-L1/APC (Biolegend, clone 29E.2A3, 1:200) and HLA-A/B/C –Alexa Fluor488 (1:100, Biolegend, clone W6/32) mouse MHC-II (I-A/I-E, 1:20, Biolegend, clone M5/114.15.2). DAPI was used as a viability dye. Samples were analysed on an Aria III laser system (BD Biosciences).

### Phospho-flow cytometry

Melanoma cell lines were treated with Accutase (EMD Millipore, #SCR005) for 10 min at 37 °C to dissociate them from the plate. Dissociated cell lines were rested at 37 °C in a CO_2_ incubator for 30 min before stimulation. After resting, cells were stimulated by adding IFNγ (Cell Signaling Technology) at a final concentration of 100 ng ml^−1^. During signalling, cells were kept in a 37 °C CO_2_ incubator. After 15 min of signalling, cells were fixed for 10 min at room temperature with a final concentration of 1.6% paraformaldehyde (Electron Microscopy Services). Cells were then pelleted, and permeabilized by resuspension in 2 ml of methanol and stored over night at −20 °C. Flow cytometry was performed using the following antibodies: HLA-DR/BV421 (BD Horizon, clone G46-6, 1:40), p-STAT5/PE-Cy7 pY694 (BD Phosflow, clone 47, 1:10) and p-STAT1/PerCP-Cy5.5 pY701(BD Phosflow, clone 4A, 1:10). Samples were analysed on a LSRII system (BD Biosciences).

### Immunohistochemistry

For HLA-DR (Santa Cruz Biotechnology (sc-53319), 1:1,000)/SOX10 (LsBio (LS-C312170), 1:30), HLA-DR-DP-DQ-DX (Santa Cruz Biotechnology (sc-53302), 1:1,000)/SOX10, HLA-A (Santa Cruz Biotechnology (sc-365485), 1:1,300)/SOX10 and PD-L1 (Cell Signaling Technology #13684,1:500)/SOX10 dual IHC, tumour sections were stained overnight at 4 °C with both antibodies. Antigen retrieval was performed using Citrate Buffer (pH6) using a Biocare Decloaking Chamber. The visualization system utilized was MACH2 (Biocare) using DAB (Dako) and Warp Red (Biocare), and counterstained with hematoxylin.

For CD4 and CD8 staining, slides were placed on a Leica Bond Max IHC stainer. All steps besides dehydration, clearing and coverslipping are performed on the Bond Max. Heat-induced antigen retrieval was performed on the Bond Max using their Epitope Retrieval 2 solution for 20 min. Slides were incubated with anti-CD4 (PA0427, Leica, Buffalo Grove, IL) or anti-CD8 (MS-457-R7, ThermoScientific, Kalamazoo, MI) for 1 h. The Bond Polymer Refine detection system was used for visualization. CD4 and CD8 were scored as per cent of infiltrating CD4(+) or CD8(+) cells in the tumour area.

### HLA-DR scoring determination

Two pathologists (M.V.E. and R.S.) who were unaware of clinical response data made independent visual estimations of the percentage of tumour membrane-specific positivity for HLA-DR, in SOX10(+) nuclei areas, in the whole-tumour section focusing at the tumour hot spots. For all staining batches positive and negative controls (human tonsil; HLA-DR is positive in germinal and non-germinal centre cells and negative in squamous epithelial cells) were included and stained appropriately and reproducibly in all cases. Furthermore, nearly all cases had positive-staining stromal cells (presumably B-cells and macrophages) as an internal control. In concordant cases (both investigators scored as ‘negative' (<5% of all tumour cells in the entire tissue section staining positive; that is, all analysable fields of view) or ‘positive' (≥5% of tumour cells in the entire tissue section staining positive; that is, all analysable fields of view), the result was averaged. For discordant cases (that is, positive versus negative interpretation, or any concerns on evaluable nature of the specimen) the investigators reviewed the case together to reach a final conclusion or consensus. If no consensus could be agreed upon, the sample was listed as non-evaluable.

### CCLE analysis

Gene-expression data (Affymetrix hg133plus2) from the CCLE were downloaded from the Broad Institute (http://www.broadinstitute.org) and analysed in R (http://www.r-project.org/)^45^. RMA-normalized melanoma cell line data were collapsed to the gene level and filtered using the ‘genefilter' package. Differentially expressed genes were identified using a *t*-test with a FDR correction^46^. Hierarchical clustering was performed using 1-Spearman's rank correlation and complete linkage. GSA was performed using the GSA package in R and the maxmean statistic^47^. Gene sets in the molecular signatures database curated gene sets C2 collection (version 3.0) were utilized for GSA.

### Cell and tumour culture

SKMEL28 and WM115 cell lines were obtained from Dr Kimberly Dahlman (Vanderbilt University), CHL-1 and HMCB melanoma cell lines were obtained from the laboratory of William Pao (Vanderbilt University). Cell line nature was not directly authenticated, but protein-marker expression was consistent with published *HLA-DRA* mRNA expression patterns (CCLE). Cell lines were confirmed mycoplasma-free and cultured in DMEM containing 10% FBS. Stimulation with recombinant human IFNγ (R&D Systems) was performed at 100 ng ml^−1^. For PDX models and *ex vivo* organotypic culture, tumours were freshly resected and sectioned using an Alto tissue matrix sectioner (Roboz Surgical, Gaithersburg, MD).

### Patients

Patient samples and data were procured based on availability of tissue and were not collected according to a pre-specified power analysis. All patients provided informed written consent on IRB-approved protocols (Vanderbilt IRB #030220 and #100178). Tumour samples for the TMA and for the HLA-DR staining cohort were obtained from tumour biopsies or tumour resections obtained for clinical purposes. Samples were obtained within 2 years of start of anti-PD-1/PD-L1 therapy (nivolumab, pembrolizumab and MPDL3280a). Only patients with available tumour samples and evaluable responses were included. In cases where multiple tissues were available for the same patient, the evaluable sample collected closest to PD-1 therapy was used for scoring. Clinical characteristics and objective response data were obtained by retrospective review of the electronic medical record. All responses were investigator assessed, RECIST defined responses or (in a single case) prolonged stable disease with clinical benefit lasting >3 years.

For the validation set, all patients were consented to an IRB-approved tissue banking protocol (for MGH patients as part of either Dana Farber Harvard Cancer Center protocols 02-017 and 11-181). Samples were obtained before therapy with anti-PD-1/PD-L1 monoclonal antibodies for research (as opposed to clinical) purposes. A linked database was prospectively maintained and regularly updated with clinical characteristics, response to therapy, date of progression (if applicable) and date of death or last follow-up visit.

### Statistical analysis

The tests of hypotheses concerning between two groups comparisons were completed using either two-sample Student's *t*-test or non-parametric Wilcoxon's rank sum test for continuous variables of interest. The analysis of variance with Tukey's multiple comparison adjustment was used for comparisons of more than two independent groups. Dichotomous data were compared using the *χ*^2^-test with the Yates correction or Fisher's exact test when appropriate. The Kolmogorov–Smirnov test (KS-test) was used to determine if the distribution of the data sets differed significantly. For PFS analysis, the survival curves were estimated using the Kaplan–Meier method with the log-rank test to examine the statistically significant differences between study groups. For gene analysis, the FDR-adjusted Student's *t*-test was used to identify the ‘winner genes' then followed by the complete linkage cluster analysis based on 1-Spearman's correlation. Statistical analyses were performed using R or GraphPad Prism. All *P* values reported were two-sided.

## Additional information

**How to cite this article**: Johnson, D. B. *et al*. Melanoma-specific MHC-II expression represents a tumour-autonomous phenotype and predicts response to anti-PD-1/PD-L1 therapy. *Nat. Commun*. 7:10582 doi: 10.1038/ncomms10582 (2016).

## Supplementary Material

Supplementary Information Supplementary Figures 1-9 and Supplementary Tables 1-4.

Supplementary Data 1 Genes altered in MHC-II (+) melanoma cell lines versus MHC-II (-) cell lines

## Figures and Tables

**Table 1:** Clinical characteristics of patients treated with anti-PD-1/PD-L1*.

	Number	Percentage
Age	56 (median)	27–81 (range)
*Gender*		
Male	16	53
Female	14	47
		
*Stage*		
M1a	3	10
M1b	4	13
M1c	23	77
LDH elevated	12	40
		
*Mutation*		
BRAF V600	6	20
NRAS Q61	7	23
BRAF/NRAS wild type	17†	57
		
Prior therapies	1 (median)	0–3 (range)
IL-2	5	20
Ipilimumab	14	47
BRAF±MEK inhibitor	4	13
Cytotoxic chemotherapy	5	17

^*^Discovery cohort, *n*=30.

^†^NRAS status unknown in two patients.
