# Supplementary material for: Melanoma-specific MHC-II expression represents a tumour-autonomous phenotype and predicts response to anti-PD-1/PD-L1 therapy
Source: Nat Commun. 2016 Jan 29;7:10582. doi: 10.1038/ncomms10582 (PMC4740184; doi:10.1038/ncomms10582)
Supplement: Supplementary Information — Supplementary Figures 1-9 and Supplementary Tables 1-4. [file ncomms10582-s1.pdf]

Supplementary Figure 1

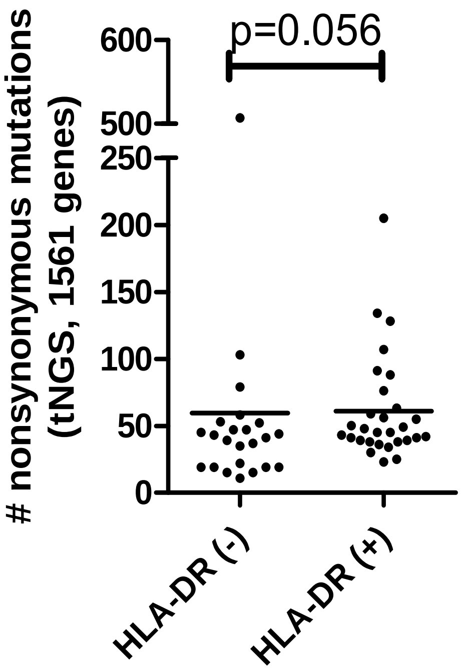

Supplementary Figure 1: HLA-DR(+) melanoma cell lines are associated with a higher mutational burden. CCLE melanoma cell lines (n=61) plotted against total expressed mutational burden. Number of non-synonymous mutations was determined by targeted sequencing of 1561 genes and these data and associated information are available in the CCLE project through the cBio portal (http://www.cbioportal.org/).

Supplementary Figure 2

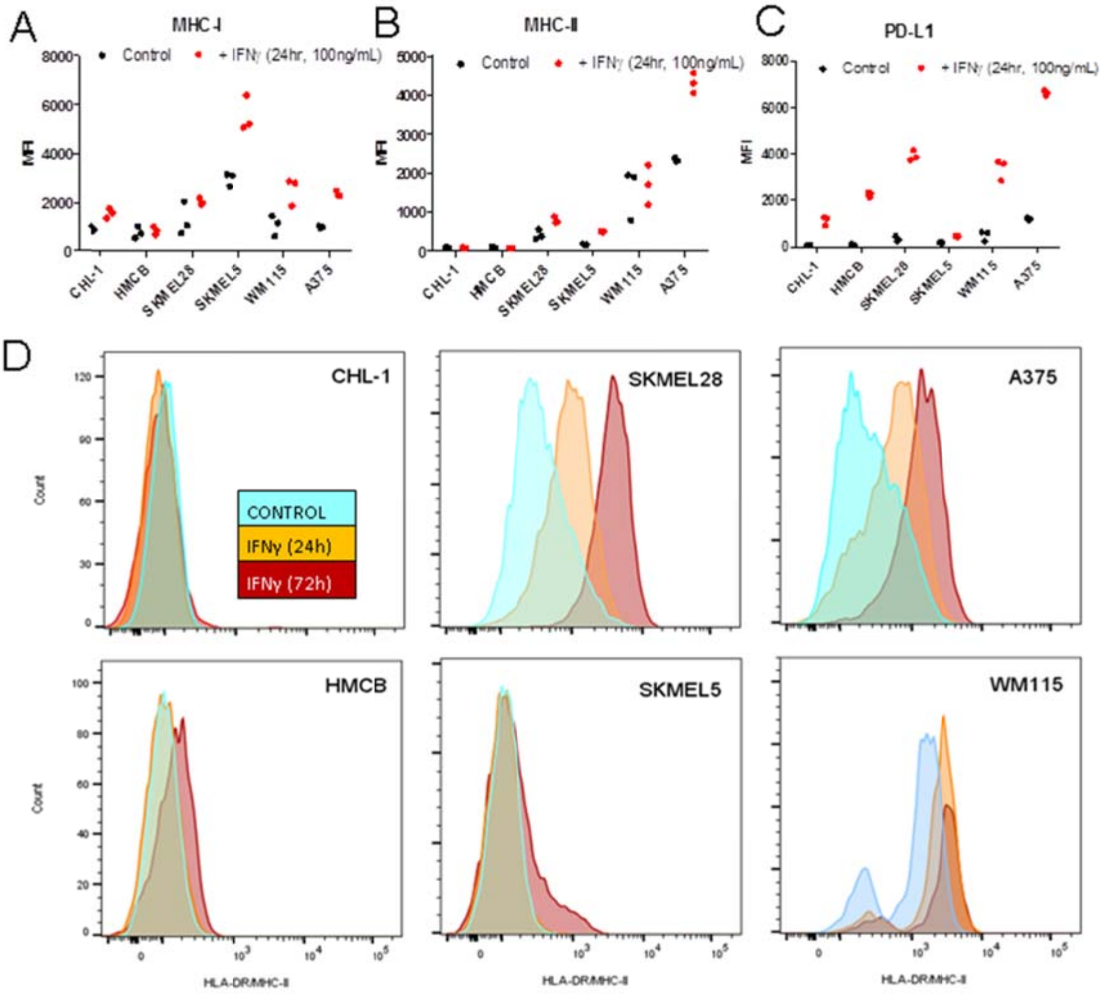

Supplementary Figure 2: Mean expression levels of MHC-I, MHC-II, and PD-L1 at baseline and after IFNγ stimulation. Melanoma cell lines were treated with 100ng IFNγ for 24hr (shown as percent positive in Fig. 2A-C) prior to harvest and live-cell staining and flow cytometry analysis for MHC-I/HLA-A/B/C (A), MHC-II/HLA-DR (B) and PD-L1 (C). Bars represent mean fluorescence intensity ± SEM for 3 experiments. (D) Histograms of HLA-DR surface expression over an extended (24-72hr) IFNγ treatment, as assessed by flow cytometry.

Supplementary Figure 3

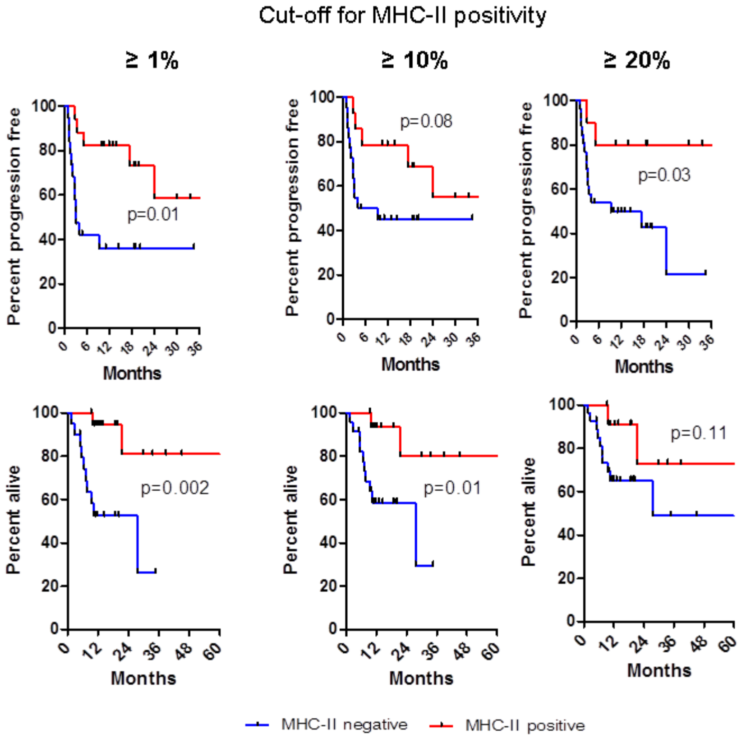

<u>Supplementary Figure 3: Progression-free and overall survival as a function of MHC-II positivity cut-points.</u> Statistical significance of PFS (top) and OS (bottom) were assessed by the log-rank statistic using different cut-points for HLA-DR positivity (1%, 10%, and 20% of tumor cells positive).

Supplementary Figure 4

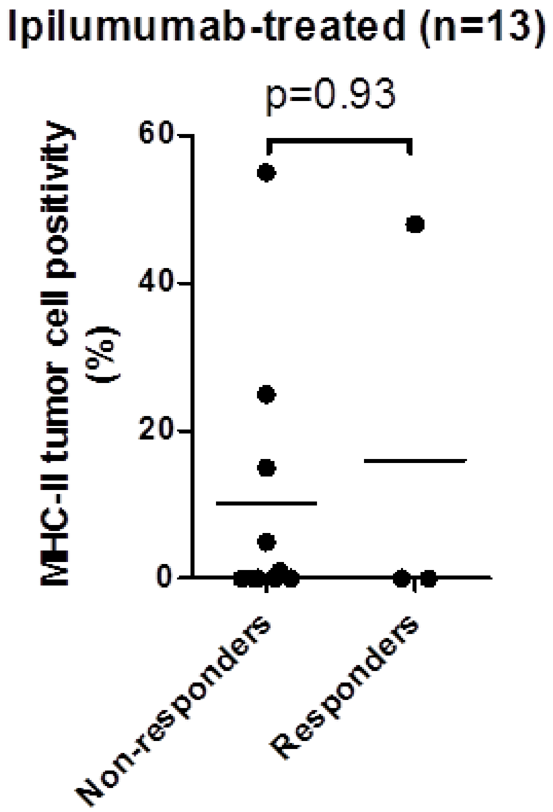

Supplementary Figure 4: MHC-II/HLA-DR positivity is not associated with ipilumumab response. Tumor membrane-specific HLA-DR expression quantified by IHC in excisional samples from patients (n=13) treated with ipilumumab (after tissue collection) were compared to treatment response. P-value represents the result of the Wilcoxan rank sum test for all responder groups versus non-responders (PD).

Supplementary Figure 5

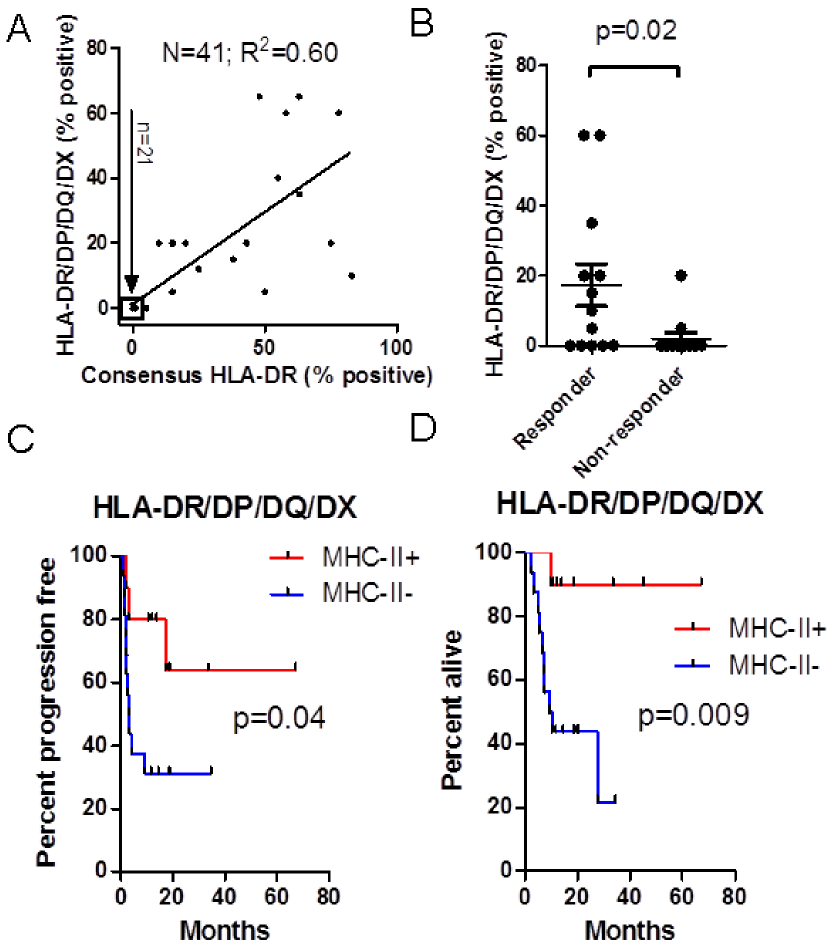

Supplementary Figure 5: High correlation of staining for two independent monoclonal antibodies for MHC-II in melanomas. A) Forty-one (41) melanoma sections were co-stained for HLA-DR and SOX10 or HLA-DR/DP/DQ/DX and SOX10 and percent of tumor cells in the entire section with MHC-II(+) membranes were calculated. There was a high degree of concordance between staining for the two antibodies. There are 21 data points at (0,0). B) HLA-DR/DP/DQ/DX positivity was used to test for association with clinical response as described for Figure 5A-B. P-value is the result of the Wilcoxan rank sum test. C) and D) PFS and OS respectively, in 26 patients (Discovery set only, non-evaluable stains excluded) discriminated on MHC-II (HLA-DR/DP/DQ/DX positivity, using a 5% cut-point (5% of total tumor cells staining positive on the entire section; no tumors stained between 1-5%). P-value represents the result of the log-rank test.

Supplementary Figure 6

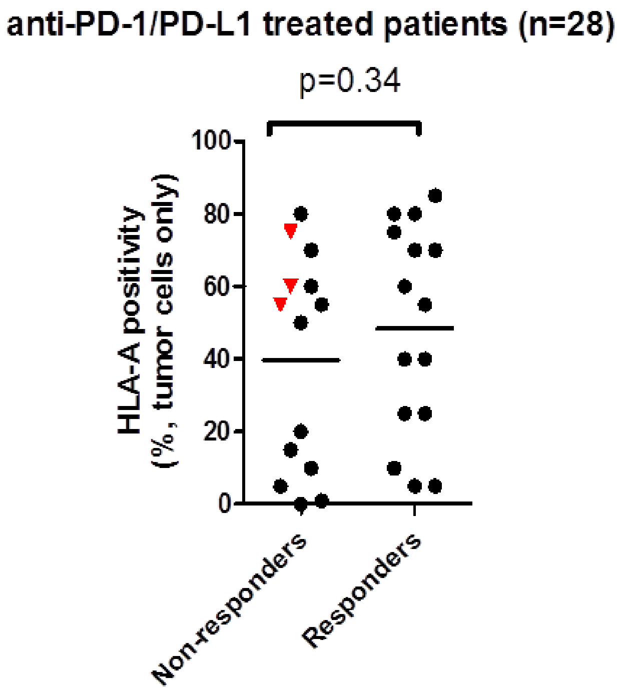

Supplementary Figure 6: MHC-I/HLA-A positivity is not associated with PD-1/PD-L1 targeted therapy response. Tumor membrane-specific HLA-A expression quantified by IHC in excisional samples from patients treated with PD-1/PD-L1 targeted therapy (after tissue collection) is compared to treatment response P-value represents the result of the Wilcoxan rank sum test for all responder groups versus PD. Mixed responders (n=3) are noted by a red triangle.

Supplementary Figure 7

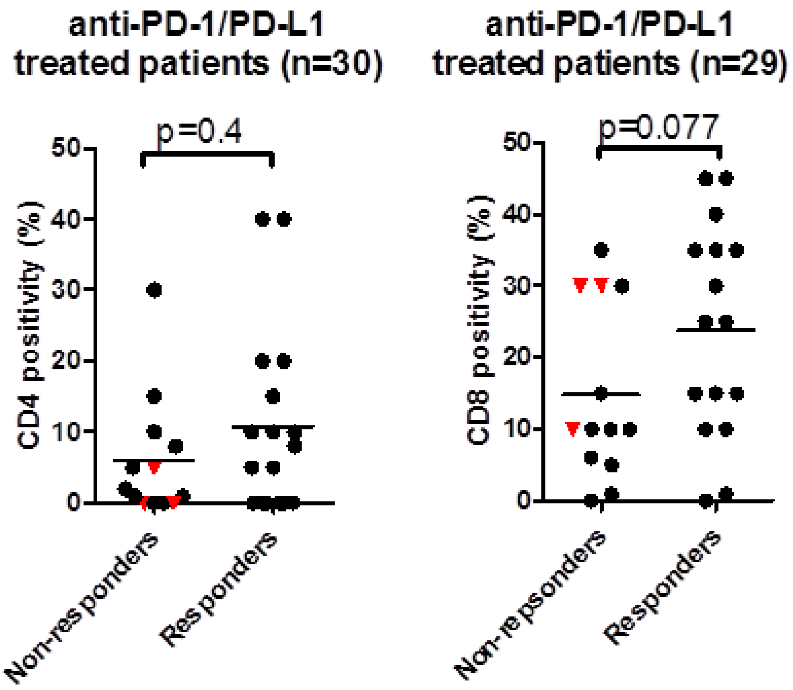

Supplementary Figure 7: CD4 positivity is not associated with PD-1/PD-L1 targeted therapy response. Tumor-infiltrating CD4(+) (left) and CD8(+) (right) cells quantified by IHC in excisional samples from patients treated with PD-1/PD-L1 targeted therapy (after tissue collection) is compared to treatment response. P-values are result of a Wilcoxan rank sum test for all responder groups versus PD. Mixed responders (n=3) are noted by a red triangle.

Supplementary Figure 8

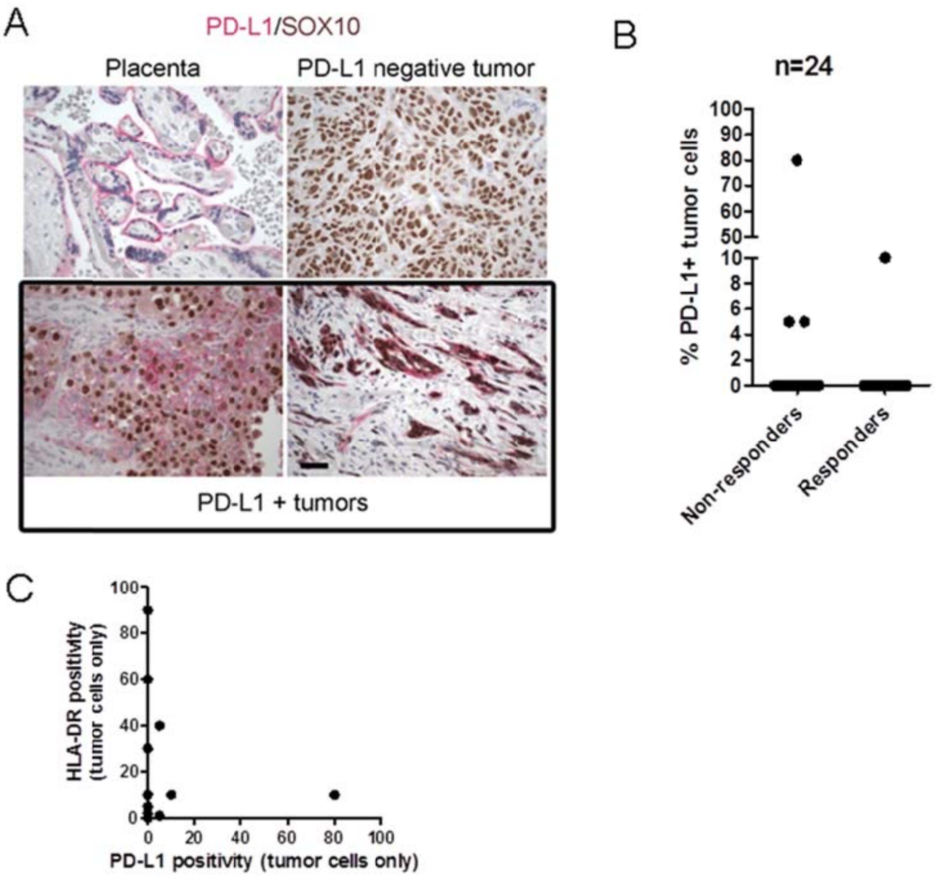

Supplementary Figure 8: Lack of PD-L1 staining with response to PD-1/PD-L1 targeted therapy. A) Representative immunostaining for SOX10 (brown/DAB) and PD-L1 (pink/Warp Red) in human placenta (positive control), a PD-L1 (-) tumor, and two PD-L1 (+) tumors. Scale bar: 50 μm B) Lack of association of PD-L1 positivity with response in a series of 24 anti-PD-1/PD-L1-treated melanoma patients. Only 4/24 patients had PD-L1 positivity noted in the tumor compartment. C) Lack of correlation between tumor cell positivity of PD-L1 and HLA-DR by IHC staining.

Supplementary Figure 9

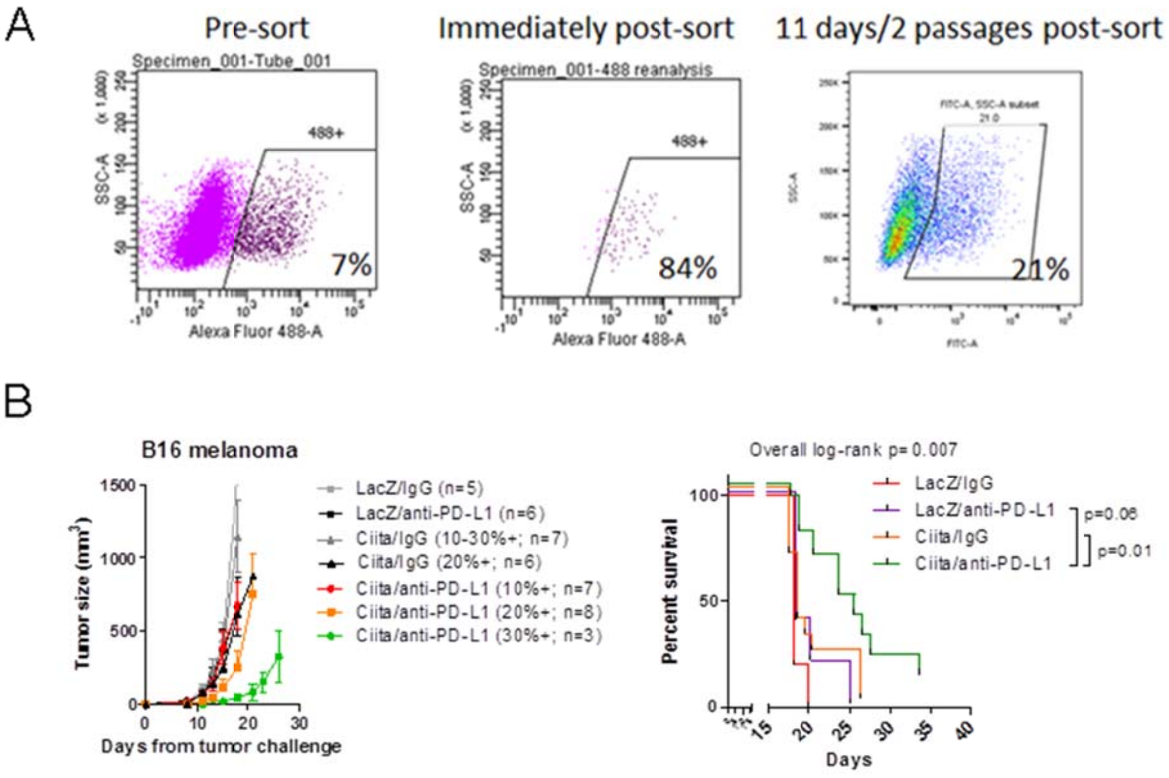

Supplementary Figure 9: Constitutive expression of MHC-II is selected against in B16 cells, but may have a functional role in response to anti-PD-L1 targeted therapy. A) Flow cytometry sorting of B16/F0 melanoma cells (anti-IA/IE) after lentiviral transduction with mouse Ciita. LACZ was used as a control for lentiviral transduction. After sorting, the percent of MHC-II+ cells was rapidly selected against in culture, despite negative selection with puromycin. B) Lentivirally-transduced cells (50,000 LACZ or Ciita) were injected subcutaneously into the flanks of C57/BL6 mice, which were subsequently treated twice weekly with 100μg/100μL anti-mouse PD-L1 mAB (BioXcel) intraperitoneally beginning on day 1 after tumor challenge. For tumor challenge, 3 separate experiments were performed for Ciita+ cell injections (assessed by flow cytometry at the day of injection as containing 10, 20, or ~30% MHC-II/IA/IE+ cells). Tumor volume was measured thrice weekly. Survival curves combined all cohorts of Ciita+ injected mice. Tumor ulceration or tumors exceeding 1000mm^3 was used as an endpoint for survival.

Supplementary Table 1: Association of HLA-DR staining on melanoma tissue microarray with clinical variables (N = 66)

	HLA-DR (+)	HLA-DR (-)	P value
	N = 20	N = 46	
Age (average, years)	57.1	61.0	0.323
Gender			
Male	12 (60%)	31 (67%)	0.562
Female	8 (40%)	15 (33%)	
Stage at resection/biopsy			
I-II	2 (10%)	6 (13%)	0.755
III	6 (30%)	17 (37%)	
IV	12 (60%)	23 (50%)	
LDH Elevated	2 (10%)	10 (22%)	0.149
Mutation			
$BRAF^{V600}$	3 (15%)	10 (22%)	0.485
$NRAS^{Q61/G12/G13}$	6 (30%)	8 (17%)	
BRAF/NRAS wild type	11 (55%)	28 (61%)	
Primary tumor ulceration	7 (35%)	15 (33%)	0.124*
Metastatic disease	18 (90%)	35 (76%)	0.192
Liver involvement#	2 (11%)	14 (40%)	0.030
Lung involvement#	10 (56%)	24 (69%)	0.349
Brain involvement#	7 (39%)	8 (35%)	0.220
Median survival	35.0 mo	35.0 mo	0.950
95% confidence interval	4.3 – 65.7 mo	0 – 78.2 mo	

*Ulceration status unknown in 20 patients #Expressed as percentage of patients with metastatic disease

Supplementary Table 2: Clinical characteristics of patients treated with ipilimumab (n=13)

	Number	*Percentage*
Age	56 (median)	34-79 (range)
Gender		
Male	8	62
Female	5	38
Stage		
M1a	1	8
M1b	2	15
M1c	10	77
LDH Elevated	5	38
Mutation		
BRAF V600	3	23
NRAS Q61	3	23
BRAF/NRAS wild type	7	54
Prior therapies	0 (median)	0-3 (range)
IL-2	0	0
Anti-PD-1/PD-L1	1	8
BRAF +/- MEK inhibitor	1	8
Cytotoxic chemotherapy	2	15

Supplementary Table 3: Concordance of HLA-DR positivity between two clinical pathologists blinded to study results (IPI and anti-PD-1/PD-L1 treated patients)

Concordance		Investigator 2 impression			
		Negative	Positive	Equivocal	Not evaluable
Investigator 1 impression	Negative	**33**	7	0	1
	Positive	5	**22**	0	0
	Equivocal	0	5	**0**	0
	Not evaluable	2	1	0	**2**

Supplementary Table 4: Consensus of HLA-DR positivity between two clinical pathologists blinded to study results (IPI and anti-PD-1/PD-L1 treated patients)

		Consensus (# of cases)		
Investigator 1 impression	Investigator 2 impression	Negative	Positive	Not evaluable
Positive	Negative	2	3	0
Negative	Positive	5	1	1
Equivocal	Positive	0	5	0
Not evaluable	Negative	0	0	2
Not evaluable	Positive	0	0	1
Negative	Not evaluable	0	0	1
